# Washed microbiota transplantation reduces glycemic variability in unstable diabetes

**DOI:** 10.1111/1753-0407.13485

**Published:** 2023-10-17

**Authors:** Yangyang Li, Qing Liu, Lingyu Zhang, Jing Zou, Rongbo He, Ying Zhou, Chen Qian, Yuxiao Zhu, Rourou Chen, Ying Zhang, Pengpeng Cai, Miao Wang, Wei Shao, Minjun Ji, Hao Wu, Faming Zhang, Zejian Liu, Yu Liu

**Affiliations:** ^1^ Department of Endocrinology, Sir Run Run Hospital Nanjing Medical University Nanjing China; ^2^ Digestive Endoscopy Center, Sir Run Run Hospital Nanjing Medical University Nanjing China; ^3^ Division of Microbiotherapy, Sir Run Run Hospital Nanjing Medical University Nanjing China; ^4^ Department of Science and Technology, Sir Run Run Hospital Nanjing Medical University Nanjing China; ^5^ Department of Pathogen Biology, Jiangsu Province Key Laboratory of Modern Pathogen Biology Nanjing Medical University Nanjing China; ^6^ Human Phenome Institute Fudan University Shanghai China; ^7^ Medical Center for Digestive Diseases the Second Affiliated Hospital of Nanjing Medical University Nanjing China; ^8^ Key Lab of Holistic Integrative Enterology Nanjing Medical University Nanjing China; ^9^ Key Laboratory of Human Functional Genomics of Jiangsu Province, Department of Biochemistry and Molecular Biology Nanjing Medical University Nanjing China

**Keywords:** fecal microbiota transplantation, glycemic variability, hypoglycemia, microbiome, unstable diabetes

## Abstract

**Background:**

Dysbiosis of gut microbiota is causally linked to impaired host glucose metabolism. We aimed to study effects of the new method of fecal microbiota transplantation, washed microbiota transplantation (WMT), on reducing glycemic variability (GV) in unstable diabetes.

**Methods:**

Fourteen eligible patients received three allogenic WMTs and were followed up at 1 week, 1 month, and 3 months. Primary outcomes were daily insulin dose, glucose excursions during meal tests, and GV indices calculated from continuous monitoring or self‐monitoring glucose values. Secondary outcomes were multiomics data, including 16S rRNA gene sequencing, metagenomics, and metabolomics to explore underlying mechanisms.

**Results:**

Daily insulin dose and glucose excursions markedly dropped, whereas GV indices significantly improved up to 1 month. WMT increased gut microbial alpha diversity, beta diversity, and network complexity. Taxonomic changes featured lower abundance of genera *Bacteroides* and *Escherichia*‐*Shigella*, and higher abundance of genus *Prevotella*. Metagenomics functional annotations revealed enrichment of distinct microbial metabolic pathways, including methane biosynthesis, citrate cycle, amino acid degradation, and butyrate production. Derived metabolites correlated significantly with improved GV indices. WMT did not change circulating inflammatory cytokines, enteroendocrine hormones, or C‐peptide.

**Conclusions:**

WMT showed strong ameliorating effect on GV, raising the possibility of targeting gut microbiota as an effective regimen to reduce GV in diabetes.

## Graphical abstract

**Highlights**

- Washed microbiota transplantation (WMT) reduces glycemic variability in patients with unstable diabetes.
- WMT alters the composition and function of gut microbiome as revealed by 16S rRNA gene sequencing and metagenomics.
- WMT led to changed profiles of fecal and serum metabolites that are correlated with improved glycemic control.

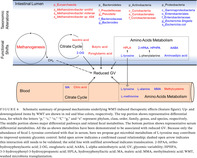

## 1 INTRODUCTION

Both type 1 (T1DM) and type 2 diabetes mellitus (T2DM) are characterized by an abnormally increased degree of glycemic variability (GV), which is manifested as swings of blood glucose levels (peaks and nadirs).^1^, ^2^ GV is inversely correlated with fasting C‐peptide levels,^3^ therefore, patients with T1DM or long‐standing T2DM are more liable to greater glycemic instability and it is always challenging to obtain optimal glycemic control in these patients.^4^ Albeit with controversies, considerable clinical evidence supports a negative role of GV in the development of diabetes complications.^5^, ^6^, ^7^, ^8^, ^9^ As a result, reduction of GV has become one of the evaluation parameters for a given treatment modality.

Lying at one extreme end of the spectrum of GV is a subgroup of patients whose metabolic control is so poor that they suffer severe instability of glycemic values with frequent and unpredictable diabetic ketoacidosis and/or hypoglycemic episodes, which is known as unstable diabetes.^10^ Although improved metabolic results have been observed with thorough education, optimized standard insulin therapy, continuous subcutaneous insulin infusion, and intraportal islet transplantation, these patients are usually refractory to attempts at restoration of glycemic control, making the search for more promising and routinely available interventional methods urgently needed.

The gut microbiota is an established regulator of host metabolism.^11^ Changing the composition of intestinal microbiota has gained much attention as a novel therapeutic modality to alleviate metabolic imbalance. Fecal microbiota transplantation (FMT) is a core therapy for correcting gut dysbiosis and has become an effective routine treatment strategy against intestinal disorders such as *Clostridioides difficile* infection.^12^ Recently, clinical studies using FMT to implant in metabolic syndrome patients of feces originating from lean healthy donors demonstrates improved insulin sensitivity and decreased glycated hemoglobin (HbA1c) levels.^13^, ^14^ The same therapeutic effects of FMT are observed by Hou's group from a randomized controlled prospective study in a Chinese cohort of newly diagnosed T2DM.^15^ Additionally, FMT halts progression of recent‐onset T1DM by preventing decline of beta cell function.^16^, ^17^ Intriguingly, multiple studies have demonstrated that gut microbiome is highly correlated with postprandial glycemic responses in both healthy adults and those with T1DM and T2DM.^15^, ^17^, ^18^, ^19^ Considering the lack of effective treatment methods for unstable diabetes, it is imperative to test whether the new method of FMT, washed microbiota transplantation (WMT),^20^ exhibits therapeutic potential to reduce GV in unstable diabetes.

This single‐arm, self‐controlled clinical trial aimed to study the effects of WMT on reducing GV in unstable diabetes. We also aimed to investigate WMT‐induced changes in the composition and function of gut microbiota and their correlations with improved GV phenotype.

## 2 METHODS

A detailed version of methodologies is available in Supplementary Methods: Data S1.

### 2.1 Human subjects

We initially screened a total of 421 patients, out of whom 17 met inclusion criteria for unstable diabetes.^21^, ^22^, ^23^ One participant withdrew due to overwhelming stress and two other patients dropped out due to poor protocol compliance. Eventually, 14 eligible participants completed the study (Table 1). Healthy donors from China Microbiota Transplantation System were screened based on published criteria.^24^ Healthy Chinese Han females (body mass index < 25 kg/m^2^, age 20–21 years) were selected. The study was approved by the Ethics Committee at Sir Run Run Hospital, Nanjing Medical University (ID: 2017‐SR‐001.S2) and conducted in accordance with the Declaration of Helsinki. The trial was registered in the Chinese Clinical Trial Registry (ChiCTR‐ONN‐17011279). All subjects gave written informed consent.

**TABLE 1:** Baseline and follow‐up characteristics of patients.

	Follow‐up time points				*p* or FDR value		
	T0	T1W	T1M	T3M	T1W versus T0	T1M versus T0	T3M versus T0
Age (years)	48.0 ± 2.9	‐	‐	‐	‐	‐	‐
Gender (F/M)	9/5	‐	‐	‐	‐	‐	‐
Duration of diabetes (years)	8.8 ± 1.4	‐	‐	‐	‐	‐	‐
Type 1 diabetes mellitus, n (%)	10 (71%)	‐	‐	‐	‐	‐	‐
BMI (kg/m^2^)	21.3 ± 0.9	‐	‐	‐	‐	‐	‐
Insulin regime, *n* (%)							
Multiple injection	13 (93%)	‐	‐	‐	‐	‐	‐
Insulin pump	1 (7%)	‐	‐	‐	‐	‐	‐
Microvascular complications, *n* (%)							
Retinopathy, *n* (%)	3 (21%)	‐	‐	‐	‐	‐	‐
Nephropathy, *n* (%)	2 (14%)	‐	‐	‐	‐	‐	‐
Peripheral neuropathy, *n* (%)	1 (7%)	‐	‐	‐	‐	‐	‐
Macrovascular complications, *n* (%)	0 (0%)	‐	‐	‐	‐	‐	‐
HbA1c, % (mmol/mol)^a^	8.1 (65) ± 0.4 (4)	‐	‐	8.5 (69) ± 0.4 (4)	‐	‐	.6182
Fasting glucose (mM)^b^	7.63 ± 1.10	8.86 ± 0.89	9.18 ± 1.00	9.72 ± 0.70	0.3458	.3458	.3458
TC (mmol/L)^b^	4.68 ± 0.25	4.44 ± 0.19	4.63 ± 0.21	4.69 ± 0.27	0.9781	.9781	.9781
TG (mmol/L)^c^	0.88 ± 0.14	1.13 ± 0.19	0.86 ± 0.11	0.81 ± 0.06	0.5348	.5770	.5770
HDL‐c (mmol/L)^b^	1.67 ± 0.14	1.64 ± 0.18	1.94 ± 0.20	2.09 ± 0.18	0.7178	**.0487** *	**.0213** *
LDL‐c (mmol/L)^b^	2.94 ± 0.23	2.45 ± 0.18	2.63 ± 0.21	2.65 ± 0.19	0.1618	.2688	.2688
C‐peptide (ng/ml)^c^	0.15 ± 0.05	0.19 ± 0.09	0.12 ± 0.05	0.09 ± 0.05	0.6085	.6114	.3141

*Note*: Values are means ± SE. *N* = 10–14.

Abbreviations: BMI, body mass index; FDR, false discovery rate; HbA1c, glycated hemoglobin; HDL‐c, high‐density lipoprotein cholesterol; LDL‐c, low‐density lipoprotein cholesterol; T1M, 1 month; T1W, 1 week; T3M, 3 months; TC, total cholesterol; TG, triglyceride.

* *p* < .05.

^a^ *p* value was calculated by Mann–Whitney test.

^b^ FDR value was calculated by a mixed‐effects model for repeated measures corrected by Benjamini and Hochberg method.

^c^ FDR value was calculated by Kruskal–Wallis test corrected by Benjamini and Hochberg method.

### 2.2 Experimental design

#### 2.2.1 Run‐in period

During the 4‐week run‐in period, patients received intensive insulin therapy in conjunction with diet and exercise interventions.

#### 2.2.2 Collection of clinical samples

Three days before WMT, patients were implanted with a continuous glucose monitor (CGM) device (MeiQi Medical Instruments Co., Ltd., Huzhou, China). One day before WMT, patients with preceding overnight 10–12 h fast had an intravenous catheter inserted into their distal arm vein. After withdrawal of baseline blood sample, they immediately went onto the steamed bun meal test (SBMT), wherein they ingested steamed buns containing 75 g of available carbohydrates. Blood samples were collected at 30 min, 1, 2, and 3 h post food ingestion. Serum, EDTA‐plasma (containing DPP‐IV inhibitors), and fecal samples were collected and stored at −80°C for later analysis.

#### 2.2.3 Fecal microbiota purification and storage

Fresh feces provided by healthy donors were processed immediately for the preparation of microbiota suspension through an automatic microbiota purification system (GenFMTer, Nanjing, China) according to the Nanjing Consensus on Methodology of Washed Microbiota Transplantation.^25^ We defined 10 cm^3^ microbiota precipitation as one basic treatment unit. At least one unit was delivered for each administration per day. The microbiota suspension was mixed with sterile glycerol to a final concentration of 10% and stored in −80°C for future use.

#### 2.2.4 WMT procedure

After bowel cleaning preparation using polyethylene glycol electrolyte solution, a mid‐gut transendoscopic enteral tube (TET) was placed in the patient's proximal jejunum through gastroendoscopy under anesthesia by certified technicians at the digestive endoscopy center. Next, the frozen fecal microbiota suspension were thawed at room temperature and delivered slowly into the mid‐gut TET tube. This WMT infusion step was repeated two more times over the next 2 days when patients were awake, using stored fecal microbiota prepared from the same donor. Patients were assigned to each donor alternatingly.

#### 2.2.5 Follow‐up

After completion of the third WMT, each patient stayed inpatient for one additional week bearing the CGM device. Then, patients were dispatched home and instructed to keep their normal routines and not make any changes to their habitual physical activity and diet, in order to avoid potentially confounding effects on gut microbiota. Their daily blood glucose and insulin dose were documented. At each follow‐up, evaluation of therapeutic effects on GV and collection of samples were conducted with identical approaches as described before WMT, except that CGM data were available only up to 1 week and HbA1c was measured only at 3 months post WMT. Ensuing safety evaluation revealed no severe or obvious adverse events.

### 2.3 Calculation of GV indices

Our primary outcomes were daily insulin doses, glucose excursions during meal tests, and GV indices calculated using published methods: mean amplitude of glycemic excursion (MAGE),^26^ SD of blood glucose (SDBG), mean blood glucose (MBG), coefficient of variation,^27^ glucose percentage time in range (TIR: 3.9–10 mmol/L),^28^ postprandial glucose excursions (PPGE), largest amplitude of glycemic excursions (LAGE), and hypoglycemic episodes (BG < 3.9 mmol/L).

### 2.4 16S rRNA gene sequencing

Our secondary outcomes were multiomics data and correlation analyzes in relation to GV phenotype. 16S rRNA gene sequencing was performed by Novogene (Beijing China) according to the workflow specified by the service provider.

### 2.5 Metagenomics sequencing and functional annotation

Metagenomics was performed by Novogene (Beijing China) according to the workflow specified by the service provider. The functional annotation to Kyoto Encyclopedia of Genes and Genomes (KEGG) database was performed through the DIAMOND software. Differential KEGG orthology (KOs) revealed by Metastats analysis were uploaded to KEGG Mapper tool (version 4.3) for enriched pathways. Network depicting connections of these pathways was constructed by Cytoscape (v 3.6.1).

### 2.6 Fecal and serum metabolomics profiling

Targeted fecal metabolomics and nontargeted serum metabolomics were performed by Metabo‐Profile (Shanghai, China). Processing of fecal and serum samples were carried out as previously described.^29^ Heatmaps with clustering showing differential metabolites were generated by Heatmapper website (http://www.heatmapper.ca/expression/).

### 2.7 Laboratory analysis

Plasma glucose, serum lipid panel including total cholesterol, low‐density lipoprotein, high‐density lipoprotein (HDL), and triglycerides were analyzed on a Cobas 8000 c701 Analyzer (Roche Diagnostics, Germany). HbA1c was measured on D‐10 Hemoglobin Testing System (Bio‐Rad, USA) using high‐performance liquid chromatography. Serum C‐peptide was detected on Cobas 8000 e602 Analyzer using chemiluminescence method. Plasma levels of enteroendocrine hormones, including glucagon‐like peptide 1 (GLP‐1; active; Millipore Cat# EGLP‐35 K), GLP‐2 (Millipore Cat # EZGLP2‐37 K), and peptide YY (PYY; Millipore Cat# EZHPYYT‐66 K) were analyzed by commercial ELISA kits. Plasma inflammatory cytokines were measured using the Meso scale discovery U‐Plex assay system with customizedly configurated panels (Meso Scale diagnostics LLC, Rockville, MD). Transforming growth factor‐β1 (TGF‐β1) was measured by commercial ELISA kit (DAKEWE Cat # DKW12‐1710).

### 2.8 Statistics

All statistical analyses were performed with GraphPad Prism, unless otherwise stated. Data are presented as either scatters, mean ± SEM, or box plot showing means with minimum and maximum values. Normal distribution of each dataset was accepted when passing both Shapiro–Wilk and Kolmogorov–Smirnov tests. For comparisons between two groups with matched datapoints and normal distribution, two‐tailed paired *t* tests were used. For comparisons between two nonmatched groups, two‐tailed unpaired *t* test or Mann–Whitney test was chosen. For comparisons among multiple groups with matched datapoints, repeated‐measures one‐way or two‐way analysis of variance (ANOVA) were adopted for data with normal distribution, whereas Friedman test or Kruskal–Wallis test were adopted for data with nonnormal distribution. All multiple comparisons were adjusted by the original false discovery rate (FDR) method of Benjamini and Hochberg method. FDR or *p* values < .05 were considered statistically significant. Spearman's correlations between metabolites and clinical parameters were calculated based on a published method,^30^ using pooled variables collected over all the follow‐up time points. The post hoc power analysis was performed via G*Power software, using one‐sided paired *t* test (*α* = 0.05, effect size = mean differences/SD; *N* = 14) as instructed.^31^

## 3 RESULTS

### 3.1 Overview of experimental design

**FIGURE 1:**
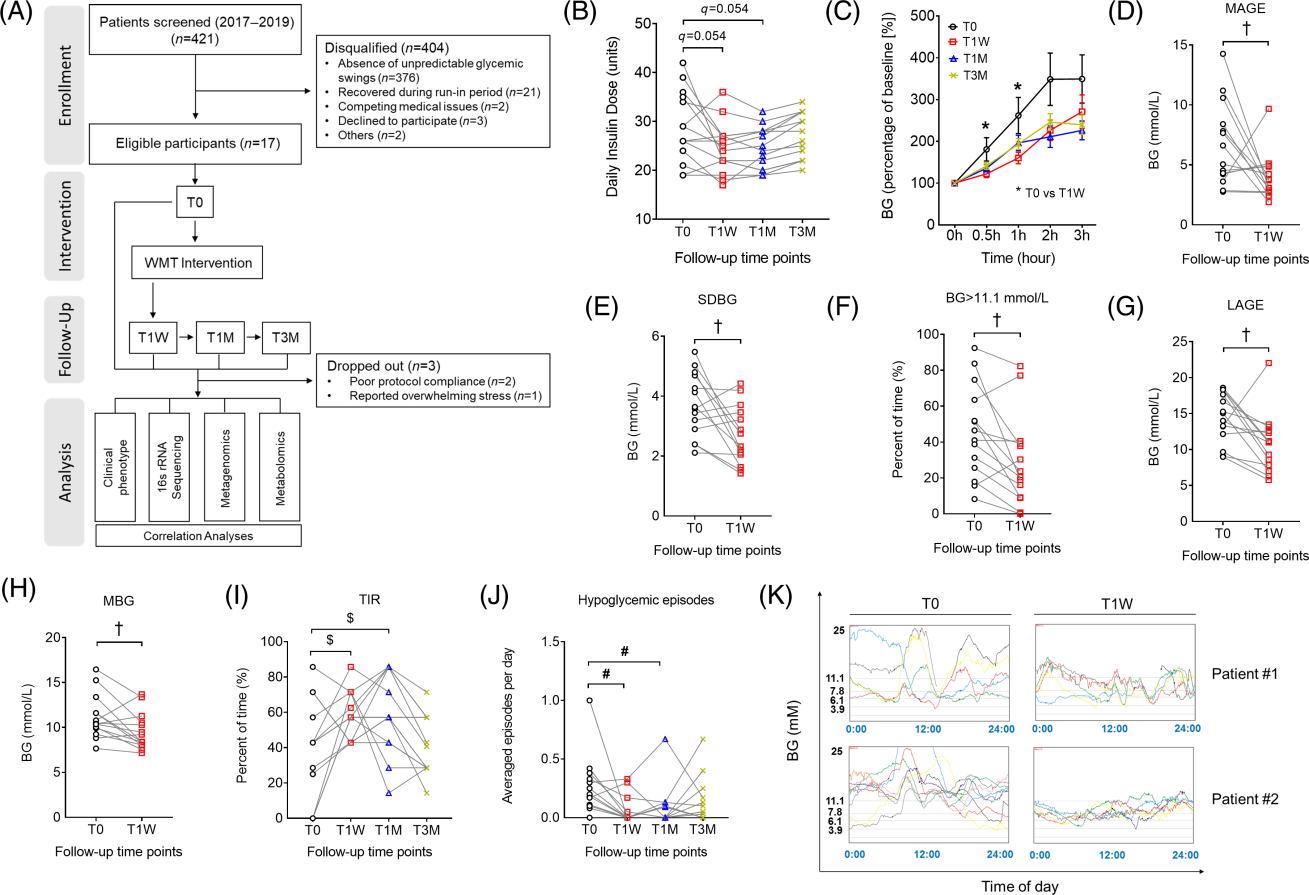
(A) Flow diagram of study design and participating patients. (B) Changes of daily insulin dose. Data of the same subject at different follow‐up time points are connected by gray lines. False discovery rate (represented as *q*‐value) was calculated using repeated‐measures one‐way analysis of variance corrected by Benjamini and Hochberg method for multiple comparisons, *n* = 14. (C) Postprandial glucose excursions expressed as percentage of baseline during steamed bun meal test (SBMT). All data are represented as mean ± SEM, *q < 0.05 by mixed‐effects model for repeated measures corrected by Benjamini and Hochberg method, *n* = 9–12. (D–H) Changes of glycemic variability (GV) indices at 1 week (T1W) calculated using continuous glucose monitor (CGM) data: (D) MAGE, (E) SDBG, (F) BG > 11.1 mmol/L, (G) LAGE, and (H) MBG. ^†^ *p* < .05 by paired *t* test (two‐tailed), *n* = 14. (I–J) Changes of GV indices at T1W, 1 month (T1M), and 3 months (T3M) calculated using self‐monitoring of blood glucose data: (I) TIR and (J) hypoglycemic episodes. ^$^ *q* < 0.05 as stated in (B); ^#^ *q* < 0.05 by repeated‐measures Friedman test corrected by Benjamini and Hochberg method, *n* = 14. (K) Representative CGM 24‐h glucose profiles demonstrating improved glycemic stability in two patients by WMT. Values of some GV indices were the same for different participants. Thus, both points and connecting lines overlapped with each other, exhibiting fewer than 14 points. LAGE, largest amplitude of glycemic excursions; MAGE, mean amplitude of glycemic excursion; MBG, mean blood glucose; SDBG, SD of blood glucose; TIR, time in range; WMT, washed microbiota transplantation.

Fourteen eligible patients with unstable diabetes (Table 1) received allogenic (healthy donors) WMT and were followed up at three time points: 1 week (T1W), 1 month (T1M), and 3 months (T3M). Their baseline parameters prior to WMT were documented (T0) and served as basis for later comparisons (Figure 1A). All patients were required to wear real‐time CGM devices from T0 to T1W.

### 3.2 WMT reduces GV in patients with unstable diabetes

Daily insulin dose dramatically reduced at T1W and T1M, but rebounded by T3M (Figure 1B). HbA1c was unaffected by the end of the study (Table 1). Notably, glucose excursions during the SBMT significantly decreased at T1W (Figure 1C). Analysis with CGM data showed that the mean amplitude of glycemic excursion, SDBG, percentage of time spent with BG > 11.1 mmol/L, LAGE, and MBG all significantly decreased (Figure 1D–H), whereas glucose percentage TIR of 3.9–10 mmol/L significantly increased (Figure S1A). Post hoc power analysis of the aforementioned CGM GV indices with a sample size of 14 revealed an averaged statistical power of 85% (Table S1), which is typically considered as an acceptable power level in multiple WMT clinical studies.^14^, ^32^ In agreement, analysis with self‐monitoring of blood glucose (SMBG) data also revealed TIR to be significantly greater at T1W and T1M (Figure 1I), suggesting the therapeutic effects of WMT could be maintained up to 1 month. Of note, hypoglycemic episodes reduced profoundly at T1W and T1M (Figure 1J). Representative 24 h CGM profiles obtained at T0 and T1W of two recipients are shown in Figure 1K. To our attention, the clinical effects of WMT on the aforementioned GV indices were not different between participants with T1DM and those with T2DM at any follow‐up time points (Figure S2). Interestingly, WMT significantly increased the level of serum HDL (Table 1), consistent with an earlier report showing that gut microbiome is associated with postprandial lipemia.^18^, ^33^ Collectively, we demonstrated a strong ameliorating effect of WMT on GV in patients with unstable diabetes. No severe WMT‐related adverse events were observed.

### 3.3 WMT alters gut microbiota composition in recipient patients

**FIGURE 2:**
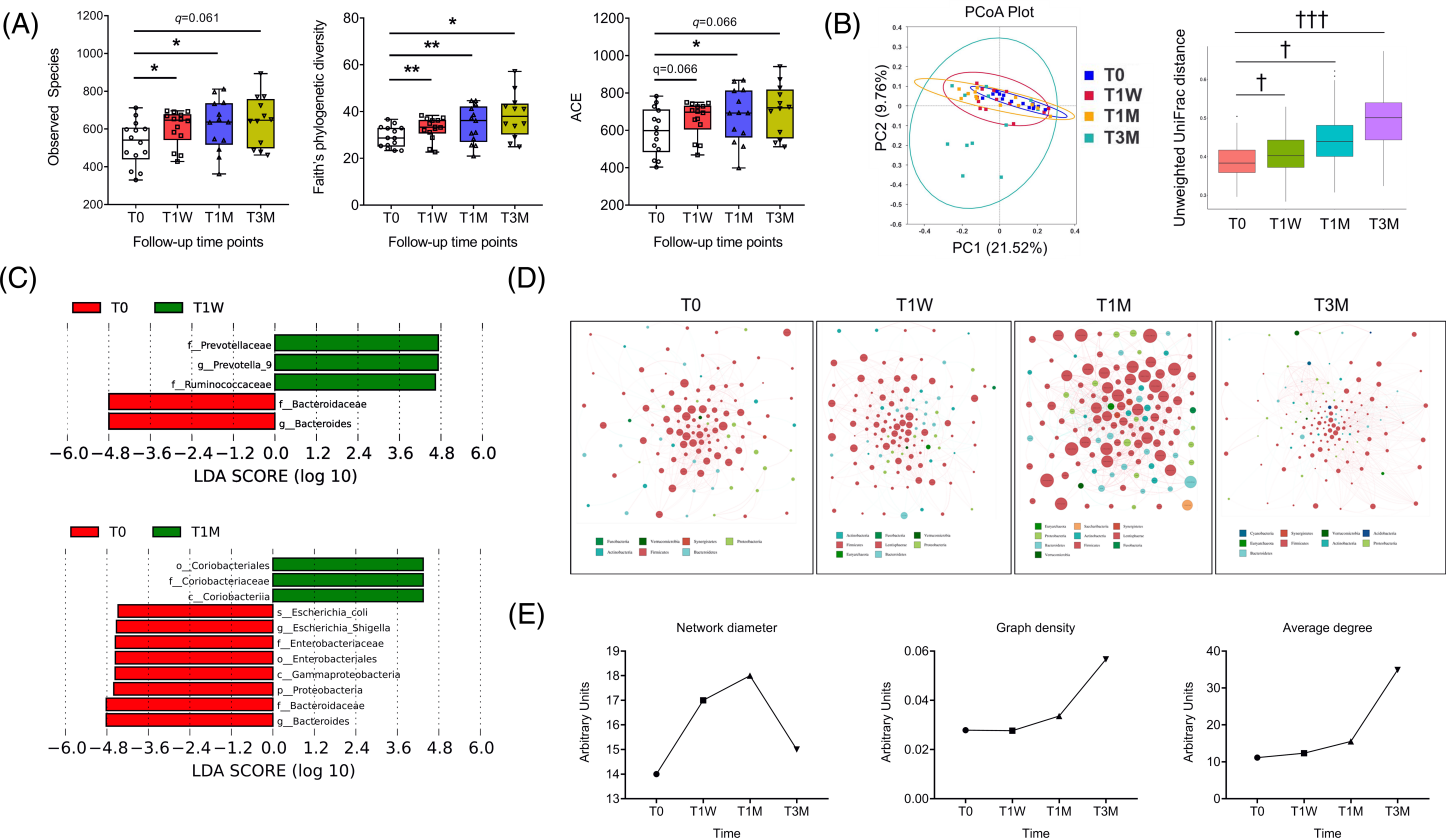
Washed microbiota transplantation (WMT) alters gut microbiota composition in recipient patients as revealed by 16S rRNA gene sequencing. (A) Comparison of alpha diversity indices, including Observed Species, Faith's phylogenetic diversity, and ACE index, between T0 and follow‐up time points. Box plots are presented with means and whiskers denoting minimum and maximum values. *q < 0.05, **q < 0.01 by ordinary one‐way analysis of variance with Benjamini and Hochberg false discovery rate correction for multiple comparisons, *n* = 12–14. (B) Comparison of beta diversity represented as PCoA that was plotted based on Unweighted Unifrac distances. Associated statistical analyzes were conducted using analysis of molecular variance, ^†^ *p* < .05, ^†††^ *p* < .001 when compared to T0. (C) Linear discriminate analysis coupled with effect size measurements of the signature differential taxa. LDA score of 4 was used as cutoff. (D) Cooccurrence network of gut microbiota showing interactions among genera. Each node represents a genus, nodes of the same color belong to one phylum, node size represents taxonomic abundance. Lines connecting nodes represent Spearman coefficients. Line thickness represents the interaction strength. Red and green lines indicate positive and negative interactions, respectively. Only significantly correlated genera with *p* < .05 are adopted to construct the network. (E) Network diameter refers to the length of the longest of all the computed shortest paths between any two nodes. Graph density refers to the ratio of the number of actual edges over that of all possible edges. Average degree refers to average number of edges per node. LDA, linear discriminant analysis; PC1, principal coordinate 1; PC2, principal coordinate 2; PCoA, principal coordinates analysis.

Next, we performed 16S rRNA gene sequencing to inspect WMT‐induced changes of gut microbial community. First, we observed an increased alpha diversity, as reflected by the Observed Species, the Faith's phylogenetic diversity, and the ACE index (Figure 2A). Similar to GV indices, alpha diversities were not different between T1DM and T2DM (Figure S3). Second, WMT changed beta diversity as represented by the principal coordinates analysis (PCoA) that is calculated using Unweighted UniFrac distances to visualize dissimilarities among all samples (Figure 2B). Scatterplot and associated analysis of molecular variance (all adjusted *p* < .05) confirmed significantly shifted clustering of gut bacterial communities by WMT. To identify signature microbial taxa driving these compositional differences, we performed LEfSe analysis (Figure 2C) and found T1W was characterized by increased abundance of genus *Prevotella* and family Prevotelleceae. At T1M, gut microbiota was enriched with family Coriobacteriaceae, order Coriobacteriales, class Coriobacteriia and was depleted with the pathogenic lipopolysaccharide‐containing species *Escherichia coli*, genus *Escherichia*‐*Shigella*, family Enterobacteriaceae, order Enterobacteriales, class Gammaproteobacteria, and phylum Proteobacteria. Both T1W and T1M were characterized by lower abundance of genus *Bacteroides* and family Bacteroidaceae. Overall, WMT‐induced changes in gut microbiota composition were reflected in specific hierarchical lineages and maintained down through constituent subtaxa. A bar graph showing differential phyla and a representative heatmap showing differential genera are displayed in Figure S4. No differences were observed between T1DM and T2DM in the relative abundance of the phylum Firmicutes or Bacteroidetes (Figure S4B,C).

Then, we built cooccurrence networks with identified genera to assess their interactions. Interestingly, the number and relative abundance of constituent genera (node size shown in Figure 2D), the number of significant connections (Table S2), and network complexity (network diameter, graph density, and average degree shown in Figure 2E) all increased after WMT, indicating a more intertwined ecological network.

### 3.4 WMT promotes divergent functional shifts in gut microbiome of recipients

**FIGURE 3:**
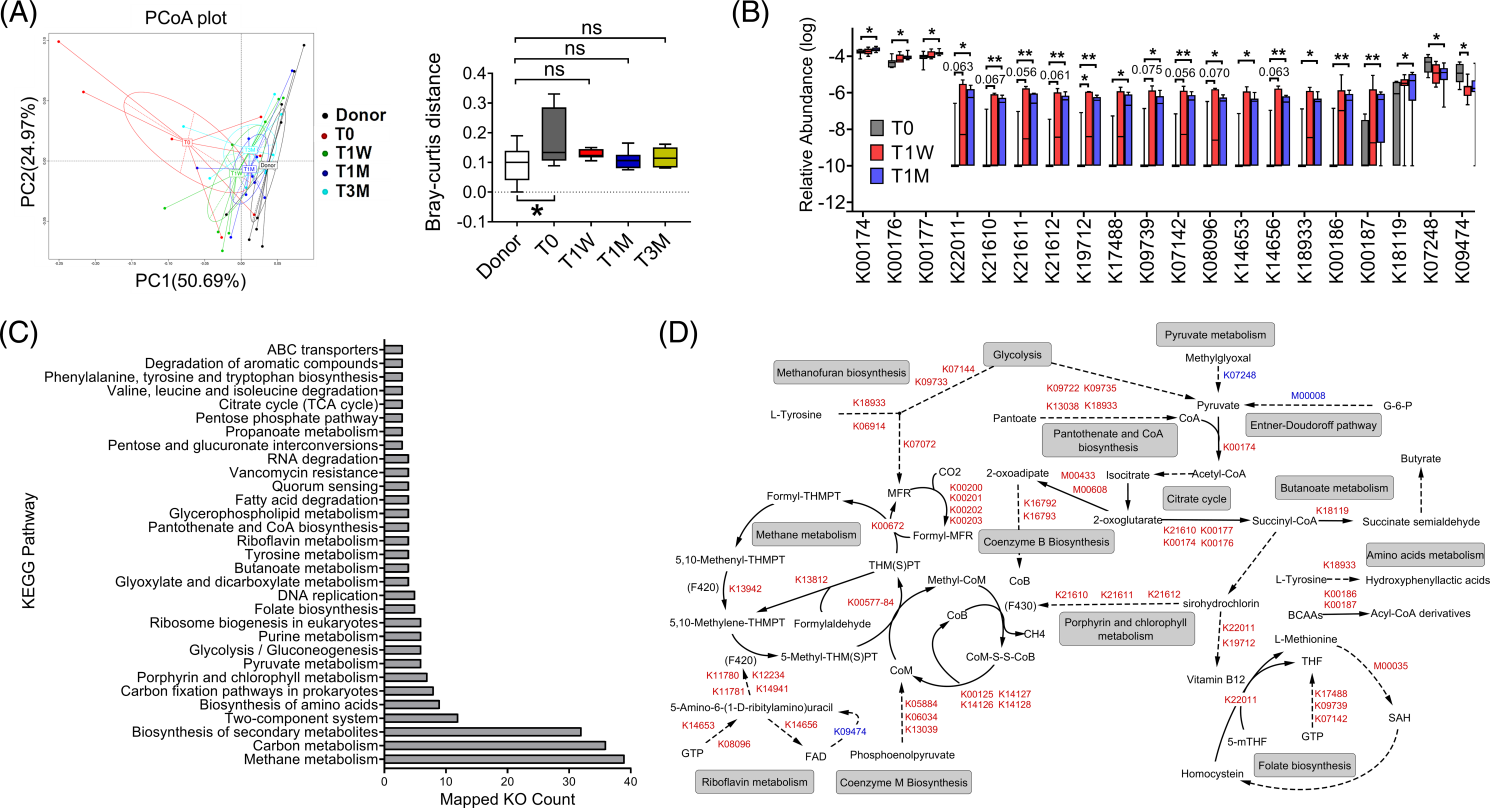
Washed microbiota transplantation (WMT) promotes divergent functional shifts in gut microbiome as revealed by metagenomics. (A) PCoA analysis of changes in the abundance of KEGG genes and associated box plots presented with means and whiskers denoting minimum and maximum values of Bray‐Curtis distance. *q < 0.05 as stated in Figure 2(A), *n* = 7–10. (B) Box plots with means showing relative changes of representing KEGG orthology at 1 week (T1W) and 1 month (T1M) in comparison to T0. Whiskers denote minimum and maximum values. Data were log transformed. *p < .05 and **p < .01 by permutation test, *n* = 7–9. (C) Enriched KEGG pathways using differential KOs. The length of bar was determined by the number of KOs mapping to each pathway. (D) Illustration of distinct metabolic pathways regulated by WMT. Differential KOs rendering each identified pathway are positioned above the reactions they catalyze. Red and blue indicate up‐ and downregulated KOs, respectively. Dashed lines indicate a series of reactions involving multiple steps. ABC, ATP‐binding cassette; KEGG, Kyoto encyclopedia of genes and genomes; KO, KEGG orthology; PC1, principal coordinate 1; PC2, principal coordinate 2; PCoA, principal coordinates analysis; TCA, tricarboxylic acid.

To further understand WMT‐induced functional changes of gut microbiome, we performed fecal shotgun metagenomics sequencing that generated a total of 1 344 921 nonredundant microbial genes, which were then annotated to KEGG KOs. WMT caused a clear and persistent shifting of microbial functional variations from T0 to become no different from those of donors (Figure 3A). Then, we focused on and mapped the 328 KOs that were initially different between donors and recipients pre‐WMT (permutation test, *p* < .05), but reverted significantly toward the levels of donors at T1W and T1M (Figure 3B; Table S3), into KEGG database for pathway enrichment analysis (Figure 3C; Table S4). Metabolic connections between as‐enriched pathways and associated biochemical reactions are depicted in Figure 3D.

The most enriched pathway was methane metabolism, an observation echoing our taxonomic results showing increased abundance of the phylum Euryarchaeota (Figure S4A), to which most methanogens belong.^34^ Second, changes in pyruvate metabolism were represented by a reduced production from methylglyoxal via *lactaldehyde dehydrogenase* (K07248) and by an augmented oxidation to acetyl‐CoA, selectively via *2‐oxoacid ferredoxin oxidoreductase* (K00174). To support, the pathway of pantothenate and CoA biosynthesis was highly enriched to promote the formation of coenzyme A, assisting pyruvate oxidation to acetyl‐CoA. With respect to the citrate cycle, WMT promoted conversion of 2‐oxoglutarate to succinyl‐CoA by upregulating *2‐oxoacid ferredoxin oxidoreductase* (K00174, K00176, K00177). Another critical finding is that metabolism of amino acids was altered, particularly for those of aromatic amino acids (AAAs) and branched‐chain amino acids (BCAAs). For example, WMT induced higher abundance of *tyrosine decarboxylase* (K18933) to promote tyrosine degradation. BCAA degradation was augmented by upregulation of *2‐oxoisovalerate ferredoxin oxidoreductase* (K00186, K00187) that unidirectionally convert the cognate α‐ketoacids of each BCAA to their corresponding CoA thiol ester derivatives. In addition, butyrate metabolism was enriched, wherein *succinate‐semialdehyde dehydrogenase* (K18119) that catalyzes formation of the precursor succinate semialdehyde from succinyl‐CoA was upregulated. Altogether, our results demonstrated WMT reshaped gut microbiome to affect metabolic pathways including methane production, pyruvate oxidation, citrate cycle metabolism, amino acid degradation, and butyrate formation.

### 3.5 WMT alters profiles of fecal metabolites

**FIGURE 4:**
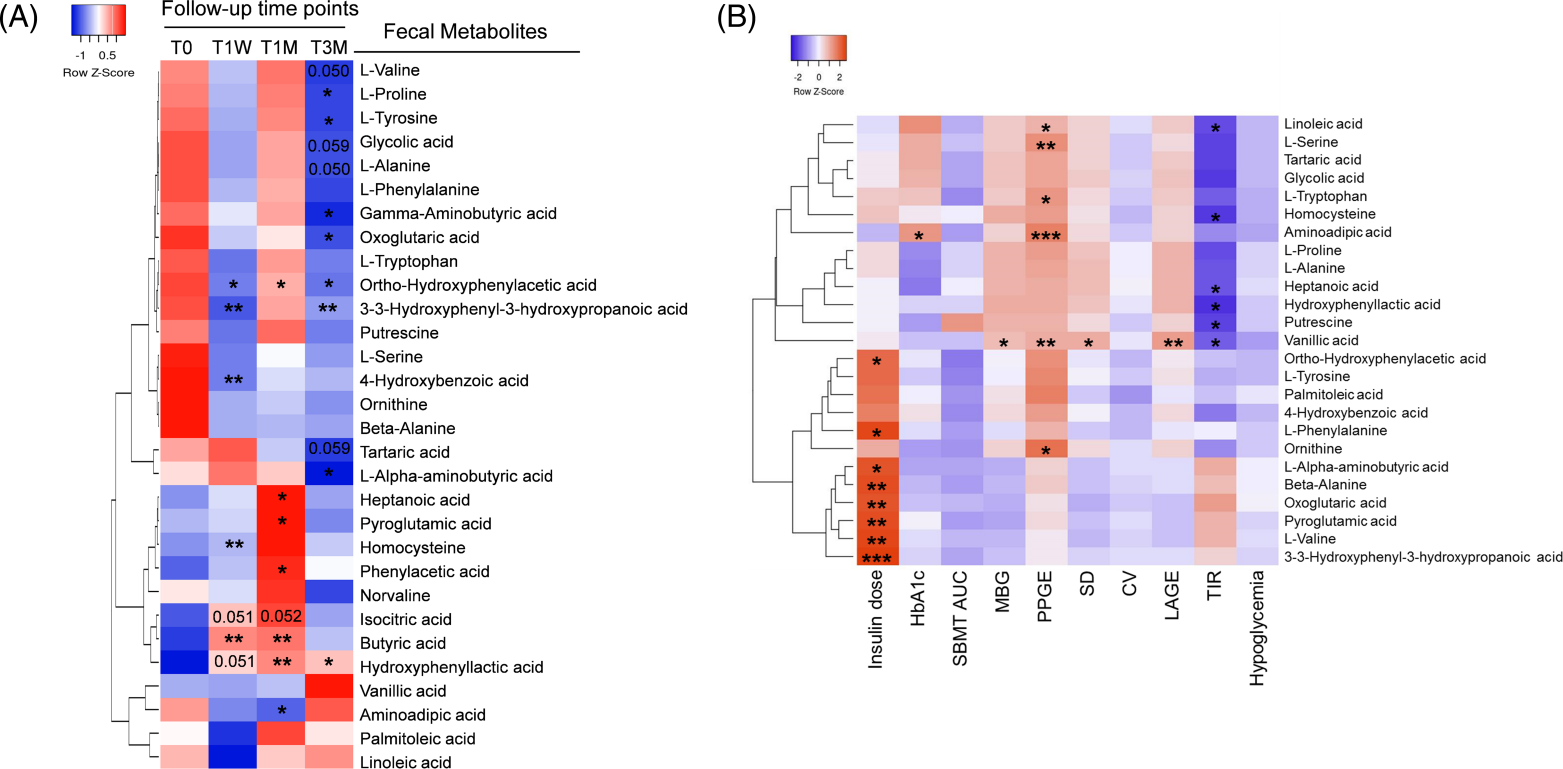
Washed microbiota transplantation (WMT) alters profiles of fecal metabolites. (A) Heatmap showing changes of fecal metabolites determined by targeted metabolomics. Colors changing from blue to red indicate higher abundance. *p* values of comparisons between T0 and each post‐WMT time point for each metabolite were calculated via either unpaired *t* test (two‐tailed) or Mann–Whitney test, depending on whether sample data passed normality test, *n* = 10–12. (B) Heatmap of Spearman's correlation coefficients between fecal metabolites and glycemic variability (GV) indices calculated using self‐monitoring of blood glucose data. Red and blue color indicates positive and negative coefficient, respectively. *p < .05, **p < .01, and ***p < .001. AUC, area under the curve; CV, coefficient of variation; HbA1c, glycated hemoglobin; LAGE, largest amplitude of glycemic excursions; MBG, mean blood glucose; PPGE, postprandial glucose excursion; SBMT, steamed bun meal test; TIR, time in range.

Next, we performed targeted fecal metabolomics to explore whether WMT‐induced compositional changes and functional shifts of gut microbiome led to alterations of metabolites (Figure 4A). Compared to T0, many metabolites derived from carbohydrate and amino acid metabolism changed in patterns generally consistent with those of genes encoding metabolic enzymes. In accordance with an altered citrate cycle pathway, isocitric acid increased at both T1W and T1M, and oxoglutaric acid decreased at T3M. For amino acid metabolism, L‐valine, L‐proline, L‐tyrosine, and L‐alanine were all downregulated at T3M, whereas hydroxyphenyllactic acid, a metabolite derived from L‐tyrosine degradation, was persistently elevated at all three follow‐up time points. In addition, 3‐3‐hydroxyphenyl‐3‐hydroxypropanoic acid and ortho‐hydroxyphenylacetic acid, two organic acids derived from L‐phenylalanine degradation, were persistently decreased. Other downregulated metabolites included L‐alpha‐aminobutyric acid at T3M, which is derived from cysteine and methionine metabolism, and aminoadipic acid at T1M, which is derived from L‐lysine metabolism. Moreover, pyroglutamic acid was increased at T1M. Butyric acid was upregulated at both T1W and T1M.

Then, we performed Spearman's correlation analysis between fecal metabolites and clinical parameters (Figure 4B; Table S5). We found the significantly downregulated BCAA, L‐valine, was positively correlated with daily insulin dose. Similar correlations were observed for other differential metabolites including 3‐3‐hydroxyphenyl‐3‐hydroxypropanoic acid, ortho‐hydroxyphenylacetic acid, L‐alpha‐aminobutyric acid, pyroglutamic acid, and the citrate cycle intermediate oxoglutaric acid. Furthermore, downregulated aminoadipic acid correlated positively with PPGE.

### 3.6 WMT alters profiles of serum metabolites

**FIGURE 5:**
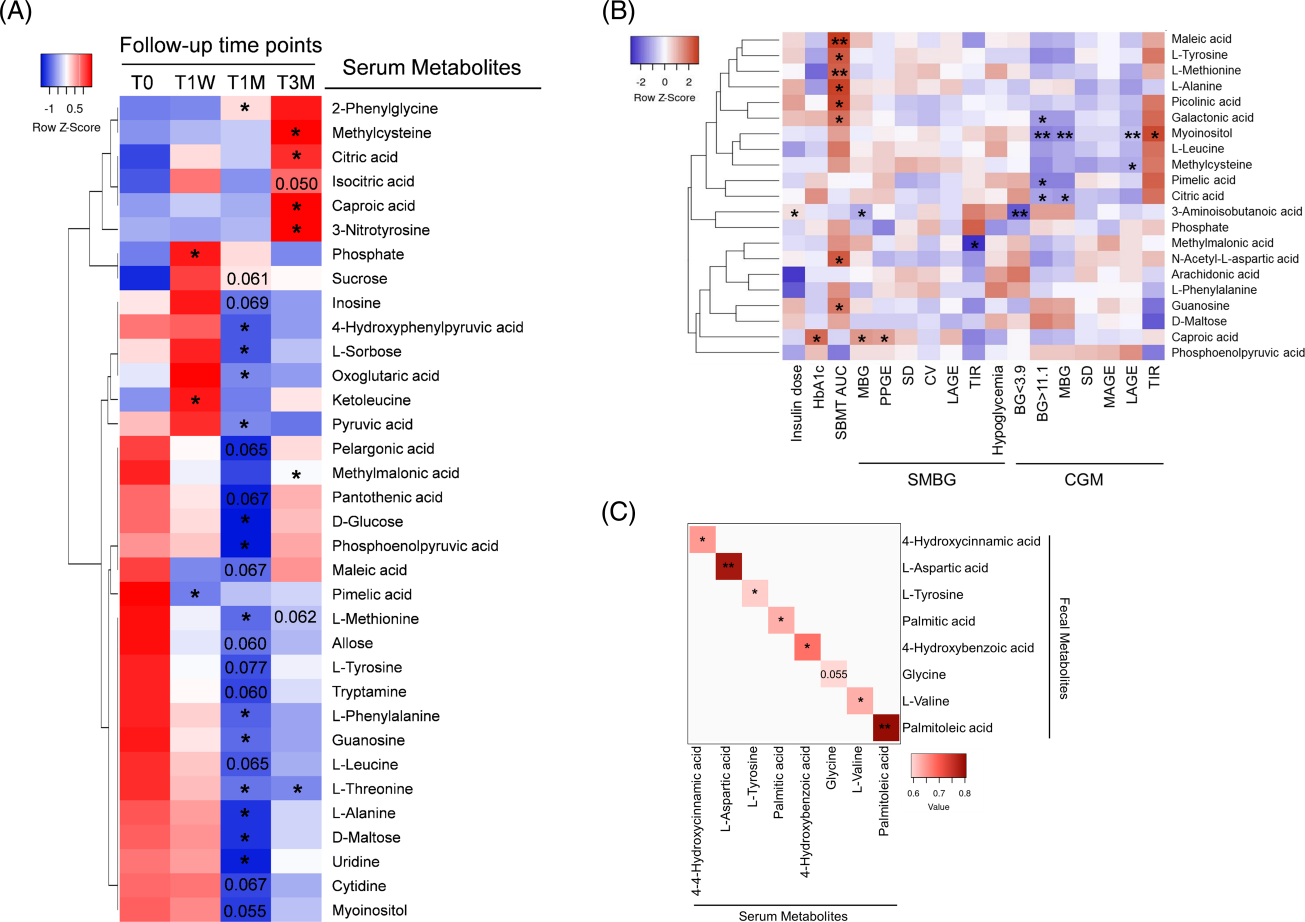
Washed microbiota transplantation (WMT) alters profiles of serum metabolites. (A) Heatmap showing changes of serum metabolites determined by non‐targeted metabolomics, *n* = 11–14. (B) Heatmap of Spearman's correlation coefficients between serum metabolites and glycemic variability (GV) indices. (C) The 8 positively correlated metabolites between fecal and serum compartments. Color range varies from light red (weaker correlation) to dark red (stronger correlation). Statistical methods were the same as stated in Figure 4. AUC, area under the curve; BG, blood glucose; CGM, continuous glucose monitor; CV, coefficient of variation; HbA1c, glycated hemoglobin; LAGE, largest amplitude of glycemic excursions; MBG, mean blood glucose; PPGE, postprandial glucose excursion; SBMT, steamed bun meal test; SMBG, self‐monitoring of blood glucose; TIR, time in range.

To gain further mechanistic insights how WMT modulates systemic glucose metabolism, we performed nontargeted serum metabolomics (Figure 5A). In alignment with an altered pyruvate metabolism, serum pyruvic acid was significantly decreased at T1M. With respect to citrate cycle, oxoglutaric acid and maleic acid were decreased at T1M, whereas citric acid and isocitric acid were increased at T3M. Consistent with the enhanced capacity of amino acids degradation, L‐methionine, L‐tyrosine, L‐phenylalanine, L‐leucine, L‐threonine, and L‐alanine were all decreased at T1M and/or T3M. In harmony, abundance of ketoleucine, the first intermediate downstream of leucine catabolism, was significantly increased at T1W. Methylcysteine and methylmalonic acid were both decreased at T3M. Altogether, serum metabolomics revealed a differential profile of circulating metabolites that genuinely agreed with changes observed in metagenomics and fecal metabolomics.

Next, we performed Spearman's correlation analysis between serum metabolites and clinical parameters (Figure 5B, Table S6). Many downregulated amino acids, such as L‐methionine, L‐tyrosine, and L‐alanine, were positively correlated with SBMT‐area under the curve (AUC). In contrast, the upregulated methylcysteine, an L‐cysteine derivative with antioxidant activity, correlated negatively with CGM‐LAGE. The downregulated methylmalonic acid correlated negatively with SMBG‐TIR. For citrate cycle intermediates, upregulated citric acid correlated negatively with CGM‐MPG and BG > 11.1 mM, whereas downregulated maleic acid correlated positively with SBMT‐AUC.

Finally, to identify serum metabolites that may have originated from the gut to exert systemic beneficial effects, we performed correlation analysis for assayed metabolites present in both feces and serum. A total of eight metabolites showed significant positive correlations (Figure 5C). Notably, L‐tyrosine was the only metabolite that was downregulated either significantly or marginally in both compartments, suggesting its microbial metabolism may contribute to improved systemic glycemic control after WMT.

### 3.7 WMT does not affect immune status or enteroendocrine hormones

Intrigued by the well‐established role of butyrate in suppressing inflammation, stimulating secretion of enteroendocrine hormones^35^ and conserving beta cell functions in T1DM,^36^ we measured serum inflammatory cytokines (interleukin [IL]‐1β, IL‐4, IL‐6, IL‐7, IL‐10, IL‐17A, IL‐18, interferon gamma, tumor necrosis factor‐α, and TGF‐β1) (Figure S5), plasma enteroendocrine hormones (GLP‐1, GLP‐2, and PYY) during the SBMT (Figure S6), and serum C‐peptide (Table 1). However, none of them was changed by WMT.

## 4 DISCUSSION

**FIGURE 6:**
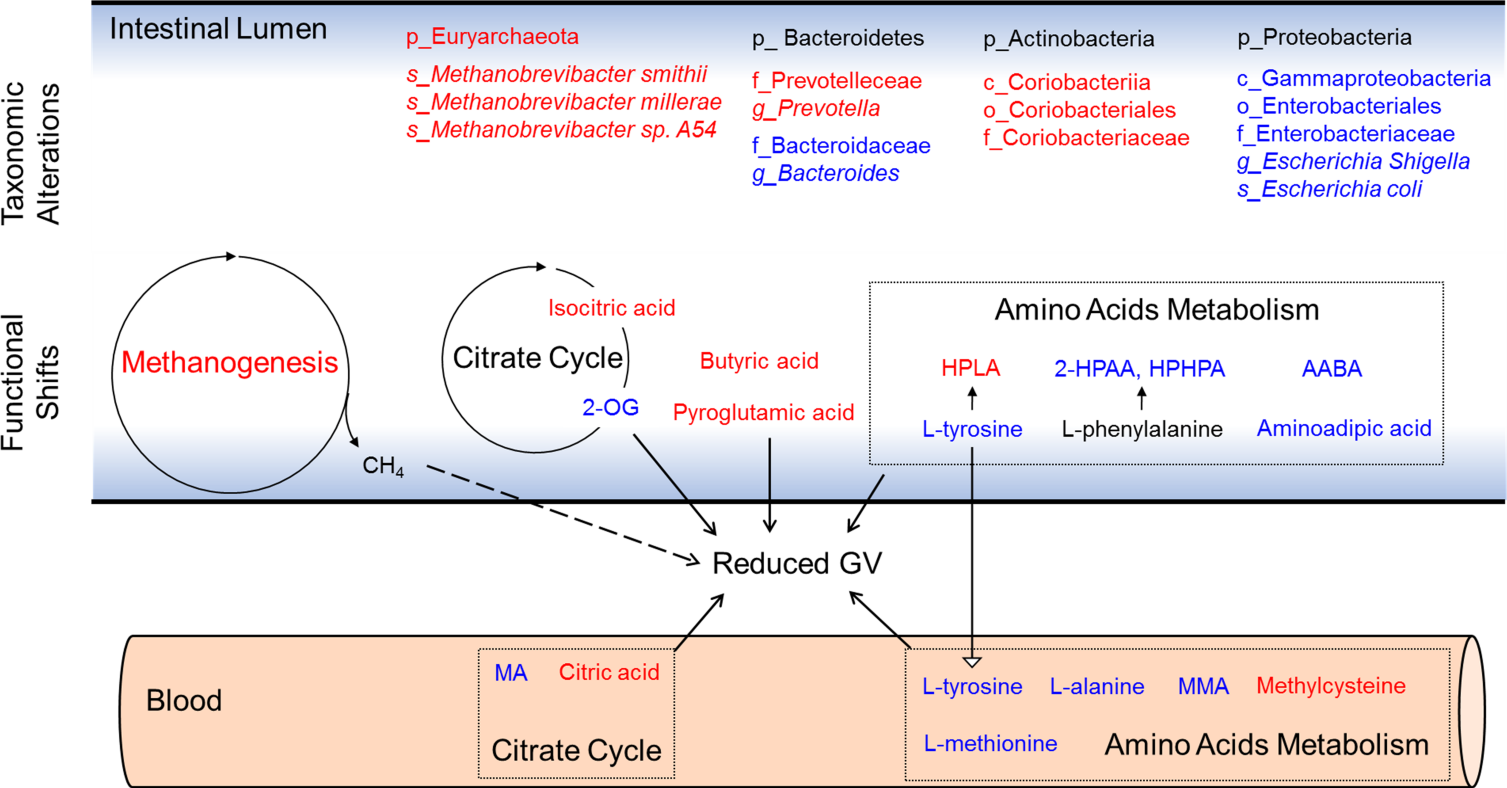
Schematic summary of proposed mechanisms underlying WMT‐induced therapeutic effects (feature figure). Up‐ and downregulated items by WMT are shown in red and blue colors, respectively. The top portion shows representative differential taxa, for which the letters “p,” “c,” “o,” “f,” “g,” and “s” represent phylum, class, order, family, genus, and species, respectively. The middle portion shows major differential pathways and related fecal metabolites. The bottom portion shows representative serum differential metabolites. All the as‐shown metabolites have been demonstrated to be associated with reduced GV. Because only the abundance of fecal L‐tyrosine correlated with that in serum, here we propose gut microbial metabolism of L‐tyrosine may contribute to improved systemic glycemic control. Solid open arrow indicates a confirmed causal relationship; dashed open arrow indicates this interaction still needs to be validated; the solid line with unfilled arrowhead indicates translocation. 2‐HPAA, ortho‐hydroxyphenylacetic acid; 2‐OG, oxoglutaric acid; AABA, L‐alpha‐aminobutyric acid; GV, glycemic variability; HPHPA, 3‐3‐hydroxyphenyl‐3‐hydroxypropanoic acid; HPLA, hydroxyphenyllactic acid; MA, maleic acid; MMA, methylmalonic acid; WMT, washed microbiota transplantation.

In this study, we report for the first time that WMT reduced GV in patients with unstable diabetes. These clinical improvements were accompanied by compositional changes of gut microbiota, leading to divergent functional shifts and altered production of relevant metabolites. Mechanistically, we identified several microbial metabolic pathways and associated metabolites as possible mediators for improved glucose homeostasis, especially of those involved in citrate cycle, amino acids metabolism and butyrate production (summarized in Figure 6). Notably, almost all the existing clinical trials showing an ameliorating effect of FMT on host glucose metabolism focused mainly on subjects with obesity and/or T2DM. Intriguingly, our participants were primarily with T1DM, thus broadening the scope of FMT in treating diabetes.

Our findings are supported by recent reports showing that FMT reduces postprandial glycemic responses in both T1DM and T2DM^15^, ^17^ but not by another study performed in diabetes with distal symmetric polyneuropathy showing that WMT fails to affect insulin dose or postprandial blood glucose.^37^ This inconsistency may be due to varied duration of diabetes, differential residual β‐cell function, fluctuation of blood glucose and infliction of distinct complications.

WMT altered several metabolic pathways that are concerned with host insulin sensitivity and glucose metabolism. For example, lower abundance of butyrate producing bacteria and decreased intestinal production of butyrate has been a well‐established characteristic for subjects with impaired glucose regulation.^38^ Reduced oxidation of pyruvate in the gut microbiota has been known associated with insulin resistance and impaired glucose metabolism.^39^ Serum level of 2‐oxoglutarate is associated with impaired glucose metabolism in obese women.^40^ Hence, our observations with increased butyrate production, enhanced pyruvate oxidation, and decreased abundance of 2‐oxoglutarate are plausible explanations to the improved glycemic control. Furthermore, WMT enhanced degradation of AAAs and BCAAs, which are well‐known contributing factors to insulin resistance. Notably, we observed a concordant pattern of changes on multiple omics levels for tyrosine degradation that led to increased production of hydroxyphenyllactic acid, a demonstrated antioxidant against reactive oxygen species (ROS). Our taxonomic results showed higher abundance of the genus *Staphylococcus* (Figure S2B), a reported producer of hydroxyphenyllactic acid.^41^ KEGG functional annotations revealed a higher level of *tyrosine decarboxylase* (K18933). Fecal metabolomics demonstrated a significantly higher amount of hydroxyphenyllactic acid. Because ROS is a contributing factor to insulin resistance,^42^ these results highlight possible involvement of this metabolite in reducing GV and warrants future research attention.

Aside from hyperglycemia, hypoglycemia was profoundly reduced by WMT in our study. Although existing literature about the effects of gut microbiota on hypoglycemia are far fewer than those on hyperglycemia, intestinal short chain fatty acids have long been recognized important in the prevention and correction of hypoglycemia in clinical trials.^43^, ^44^ Of particular interest, recent studies using germ‐free and antibiotics‐treated gut microbiota‐depleted mice demonstrated reduced content of SCFAs (including butyrate) to impair counterregulatory responses.^45^, ^46^ These studies all point toward a positive role of butyrate on reducing hypoglycemic events. Hence, our observed elevation of butyrate in patients with unstable diabetes may help explain reduced episodes of hypoglycemia after WMT intervention.

Our study is subject to three main limitations. First, due to limited number of eligible patients, a placebo or autologous microbiota was not included to rule out placebo effects. Second, animal experiments validating therapeutic effects of candidate bacterial species or metabolites are lacking. This is mainly because an animal model representing high GV in diabetes has not been developed or widely accepted, at least to our best knowledge. Third, sample size of the current study is small, making wide applicability of our findings need further validation in larger and more diverse populations, when taking into account the regional and ethnic variations in gut microbiota.

In conclusion, we showed a strong albeit short‐term effect of WMT on reducing GV in unstable diabetes. These findings provide scientific support for WMT as a clinical treatment option to restore glucose homeostasis in patients bearing glucose swings.

## FUNDING INFORMATION

This work was supported by the National Natural Science Foundation of China (82070849, 82270871, 81770778, 81800735, 81800724); the National Key R&D Program of China (2022YFA0806103); the Jiangsu Provincial Key Research Development Program (BE2022794); the Natural Science Foundation of Jiangsu Province (BK20180672); the Natural Science Foundation of the Jiangsu Higher Education Institutions (18KJB310005).

## DISCLOSURE

The authors report no conflicts of interest.

## Supporting information


**Data S1.** Supplementary methods.

**Table S2.** Network genus interactions.

**Table S3.** Differential Kyoto encyclopedia of genes and genomes (KEGG) KOs.

**Table S4.** Enriched Kyoto encyclopedia of genes and genomes (KEGG) pathways.

**Table S5.** Spearman correlation analysis between glycemic variability (GV) and fecal metabolites.

**Table S6.** Spearman correlation analysis between glycemic variability (GV) and serum metabolites.

## Data Availability

The data supporting the findings of this work are available within the article and its supplementary materials. All the original sequencing data sets will be deposited in BioProject (PRJNA826306). Currently, they are available from the corresponding author on request.
