# Supplementary material for: Washed microbiota transplantation reduces glycemic variability in unstable diabetes
Source: J Diabetes. 2023 Oct 17;16(2):e13485. doi: 10.1111/1753-0407.13485 (PMC10859319; doi:10.1111/1753-0407.13485)
Supplement: Supplementary file 1 — Data S1. Supplementary methods. [file JDB-16-e13485-s004.docx]

1. **Supplementary Methods**
   1. *Human subjects*

Patients were recruited both online (e.g. social media advertisement and APP post) and offline (e.g. in-clinic and community flyers). Target population were those finding themselves difficult to control blood glucose. Walk-in visits and recruitment mainly took place at Sir Run Run Hospital Nanjing Medical University, from May 2017 to February 2019. All patients were informed with the aim of the study, expected beneficial outcomes, and possible side effects. We initially screened a total of 421 patients interested in participating this study, out of which 17 met the defined criteria for unstable diabetes (detailed below). All those 17 patients had relied on insulin treatment for at least five years, with a glycated hemoglobin (HbA1c) less than 14% and low to absent basal secretion of c-peptide (serum level <0.6 ng/mL). They were instructed from an endocrinologist to undergo careful diabetes management for four weeks by receiving administration of either continuous subcutaneous insulin infusion or multi-dose insulin injection together with diabetes education, accompanied by frequent self-monitoring of blood glucose. Regardless of these management efforts, these patients still experienced at least two of the following syndromes that defined them as with unstable diabetes [1-3]: (i) occurrence of ketoacidosis events; (ii) occurrence of severe hypoglycemia, defined as inability of self-treatment but require another person’s assistance to recover; (iii) presence of hypoglycemic unawareness based on continuous or self-monitoring blood glucose data and Clarke score ≥4; (iv) high glycemic variability defined by at least one of the following: a mean blood glucose SD>2.22 mmol/L, MAGE>3.33 mmol/ L, and a low blood glucose index (LBGI) >5. Exclusion criteria include (i) HbAlc>14%; (ii) pregnancy, lactation, or an intent to become pregnant during the course of the study; (iii) presence or history of active infection including hepatitis B, hepatitis C, or tuberculosis; (iv) gastrointestinal disorders; (v) use of antibiotic or probiotic for >3 days within preceding 3 months; (vi) contraindications related with gastroenteroscopy; (vii) alcohol or substance abuse. During the study course, one college student patient withdrew due to reported overwhelming stress from finals and two other patients dropped out due to poor protocol compliance. Eventually, 14 eligible participants completed the study, nine females and five males, ten with type 1 diabetes and four with type 2 diabetes. Please see Table 1 for detailed characteristics of patients.

Healthy donors were recruited through advertising flyers posted on university campus. Eligible subjects were chosen based on published criteria with minor modifications [4]. Eventually, two lean, omnivorous, healthy Chinese Han females (BMI<25 kg/m^2^, age 20-21 years), with no history of gastrointestinal diseases, were selected. They had taken minimal to no medications that might interfere with gut microbiota composition in the preceding three months, such as antibiotics or probiotics. They passed screening for the absence of infectious diseases, such as HIV, hepatitis B, hepatitis C, and tuberculosis. Their feces were also screened for the absence of pathogenic parasites (Cryptosporidium, Entamoeba histolytica, Giardia lamblia, Microspore, Isosporiasis), bacteria (Clostridioides *difficile*, *Shigella spp.*, *Salmonella spp.*, *Campylobacter spp.*, *Escherichia coli O157 H7*, Shiga-producing *Escherichia coli*, *Yersinia enterocolitis*; *Aeromonas spp.*, *Plesiomonas spp.*, *Vibrio spp.*), and virus (Rotavirus, Adenovirus, HEV, and Polio virus). Donors were excluded if the above screenings showed positive.

The study was approved by the Ethics Committee at Sir Run Run Hospital, Nanjing Medical University (ID: 2017-SR-001.S2) and the trial was registered in the Chinese Clinical Trial Registry (ChiCTR-ONN-17011279). All participants gave informed written consent.

- 1. *Experimental design*

This was a self-controlled clinical trial. We studied the effect of allogenic (healthy donor) WMT on glucose variability in relation to gut microbiota composition at follow-ups of one week, one month, and three months, as compared to themselves at pre-WMT (T0) (Figure 1).

- - 1. *Run-in period*

During the four-week run-in period, the patients received intensive insulin therapy in conjunction with diet and exercise interventions. Specifically, patient and designated doctors progressively identified food, exercise, and lifestyle that could most significantly affect blood glucose control, based on which a well-defined therapeutic plan suitable for that patient's daily routine was developed. A dietician helped customize diet to the patient's habits. Management of the choice of foods and insulin therapy was reinforced by the teaching and training program on carbohydrate counting. Patients were asked to avoid intense physical activities, and to keep the total caloric content of their diet constant. Blood glucose was checked and recorded using Gold-Accu meter (Sannuo Biosensor Co., Ltd., Changsha, China) via finger pricking. Insulin doses per day were documented.

- - 1. *Collection of clinical samples*

Three days before WMT, patients were implanted with a continuous glucose monitor (CGM) device (MeiQi Medical Instruments Co.,Ltd., Huzhou, China). Afterwards, feces were collected when available and stored at -80^o^C within one hour for later analysis. One day before WMT, patients with preceding overnight 10-12 hours fast were inserted with an intravenous catheter into their distal arm vein. After withdrawal of baseline blood sample, they immediately went onto the steamed bun meal test (SBMT), wherein they ingested steamed buns containing 75g of available carbohydrates. Blood samples were collected sequentially at time intervals of 30 min, one hour, two hours, and three hours post food ingestion. For the preparation of serum, whole blood was left undisturbed at room temperature for 30 min, and then centrifuged at 2,000g for 15 min. For plasma preparation, blood was collected into ice-cooled EDTA-plasma tubes, immediately added with DPP-IV inhibitors in 30 seconds, gently mixed and kept on ice. Within one hour after collection, plasma samples were obtained by centrifugation at 2,000g, 4^o^C for 10 minutes. All samples were tested immediately or saved in -80^o^C until analysis.

- - 1. *Fecal microbiota purification and storage*

Fresh feces provided by donors were processed immediately for the preparation of gut microbiota through an automatic microbiota purification system (GenFMTer, Nanjing, China) [5]. Specifically, feces were first dispersed homogenously with normal saline at a ratio of 100g to 500ml, followed by built-in microfiltration system to remove contaminating matters such as parasite eggs, fungus, and unabsorbed remnants. The resulting infiltrates were centrifuged for 3 min at a speed of 700g to form bacterial pellet. Upon aspiration of supernatant, the pellet was resuspended and washed in saline followed by centrifugation. After being washed 5 times, gut bacterial pellet was evenly dispersed in saline. This methodology was recently coined as washed microbiota transplantation (WMT), which enables completion of the whole process from defecation to intestinal infusion of these freshly prepared bacteria in one hour. Finally, the fecal microbiota suspension was mixed with sterile glycerol to a final concentration of 10% and stored in -80℃ for future use.

- - 1. *WMT procedure*

Patients first underwent a bowel preparation using polyethylene glycol electrolyte solution prior to WMT procedures. Then, a nasojejunal tube was placed in the patient’s proximal jejunum through gastroendoscopy under anesthesia by certified technicians at the digestive endoscopy center. Next, the cryostored fecal microbiota suspension were thawed at room temperature (~50 cm^3^ fecal microbiota in ~100 ml normal saline) and delivered slowly into the mid‐gut through the implanted nasojejunal tube within ten minutes. This WMT infusion step was repeated two more times over the next two days when patients were awake, using stored fecal microbiota prepared from the same donor. Patients were assigned to each donor alternatingly; that is, every other individual was assigned to the same donor.

- - 1. *Follow-up*

After completion of the third WMT, each patient stayed inpatient for one additional week, during which time their BG levels were recorded using both a glucose meter and the born CGM device. Then, patients were dispatched home and instructed to keep their normal routines and not make any changes to their habitual physical activity and diet, in order to avoid potentially confounding effects on gut microbiota. Their daily blood glucose was monitored and recorded using glucose meters. Daily insulin doses were also documented. All patients were followed up one week (T1W), one month (T1M), and three months (T3M) after WMT treatment. At each follow-up time point, evaluation of therapeutic effects of WMT on glucose metabolism and collection of blood samples were conducted with identical approaches as described before WMT, except that CGM data were only available at T1W and HbA1c was only measured at T3M. Feces were collected in hospital during follow-up visits in an identical fashion as before WMT. In case of no defecation, patients were instructed to collect the next feces at home, temporarily store in household -20^o^C freezer and bring to our laboratory within 24 hours. Ensuing safety evaluation revealed no severe or obvious adverse events during WMT treatment and follow-up. Only one patient experienced transient diarrhea, but recovered within 24 hours spontaneously. Strategies to increase protocol adherence included provision of free glucose meters & strips, discount of treatment costs, and timely consultation.

- 1. *Calculation of glycemic variability indices*

Our primary outcomes were daily insulin doses, glucose excursions during meal tests, and various GV indices calculated from recorded glucose values. According to the criteria as previously described [6], MAGE was calculated as the arithmetic mean of blood glucose (BG) decreases from peaks to neighboring nadirs. Only differences exceeded the value of one standard deviation (SD) of the blood glucose over 24-hour period were summed up and divided by the number of eligible counts during that day. Mean blood glucose (MBG) was calculated as the arithmetic mean of BG values obtained per day. SDBG was calculated on BG values per day in Excel. Coefficient of variation (CV) was calculated as the ratio of SD to mean glucose values, expressed as a percentage [7]. Glucose percentage time in range (TIR: 3.9-10 mmol/L) was calculated as percentage (%) of CGM readings or SMBG hours spent in target glycemic range per day [8]. Postprandial glucose excursions (PPGE) was defined as the change of BG from before to after a meal. Largest amplitude of glycemic excursions (LAGE) was calculated as the maximal BG level minus the minimal BG level during each day. Hypoglycemic episodes were counted when BG <3.9 mmol/L.

- 1. *16S rRNA sequencing*

Our secondary outcomes were multi-omics data and correlation analyses in relation to GV phenotype. 16S rRNA sequencing was performed by Novogene (Beijing China) according to the workflow specified by the service provider. Specifically, bacterial genomic DNA was extracted using the CTAB/SDS method. Briefly, fecal samples were incubated with CTAB lysis buffer supplemented with lysozyme. The resulting supernatant was mixed with phenol:chloroform:isoamyl alcohol (25:24:1, v/v), centrifuged to clarify and the consequent supernatant was further mixed with chloroform:isoamyl alcohol (24:1, v/v). After centrifugation, DNA in the new supernatant was precipitated by isopropanol. DNA concentration was determined by Qubit® DNA Assay Kit using Qubit® 2.0 Fluorometer (Life Technologies, CA, USA), checked for purity by 1% agarose gels and diluted to 1 ng/µl. The V3–V4 region of 16S rRNA was amplified by PCR primers (515F-806R) to construct an amplicon sequencing library using Ion Plus Fragment Library Kit (Thermo Fisher Scientific). The amplicon was standardized and purified before being sequenced on an Ion S5^TM^ XL platform. High-quality clean reads were obtained by the Cutadapt (V1.9.1) quality control process and depleted of chimera sequences by comparing with the reference database (Silva database) using UCHIME algorithm. OTU representative sequences were assigned at 97% similarity using Uparse software (v7.0.1001) and were taxonomically classified using the Mothur algorithm trained on Silva database. Alpha-diversity indices were calculated by QIIME (V1.7.0). Principal coordinates analysis was performed by QIIME (V1.7.0) to calculate the unweighted UniFrac distance, followed by AMOVA test to detect differences between groups and finally displayed by R package (V2.15.3). The linear discriminant analysis (LDA) effect size (LEfSe) analysis was used to identify differential taxa of biological relevance between groups adopting the default LDA score set at 4. Metastat analysis was performed to assess significant differences in specific taxa between groups.

- 1. *Metagenomics sequencing*

Metagenomics was performed by Novogene (Beijing China) according to the workflow specified by the service provider. Specifically, bacterial genomic DNA was extracted using the abovementioned CTAB/SDS method. A total amount of 1 µg DNA were used to construct sequencing libraries by NEB Next® Ultra DNA Library Prep Kit for Illumina® (NEB, USA). DNA was fragmented by sonication, ends-repaired, polyA-tailed, and ligated with a sequencing adaptor for Illumina sequencing, followed by PCR amplification and purification (AMPure XP system). The insert size of library was assessed by the Agilent Bioanalyzer 2100 system and quantified by qPCR. Clustering of the index-coded samples was performed on a cBot Cluster Generation System using HiSeq 4000 PE Cluster Kit (Illumina) according to the manufacturer’s instructions. After cluster generation, the library preparations were sequenced on an Illumina HiSeq 4000 platform and 150-bp paired-end reads were generated. Adaptor and low-quality reads were discarded from the raw reads, and the remaining reads were filtered to eliminate human host DNA using Bowtie2.2.4 software. Eventually, we acquired 269.2 Gb high-quality pair-end reads from the 42 samples with an average of 6.4 Gb per sample after removing human DNA reads. All high-quality reads were assembled to Scaftigs using SOAPdenovo V2.04, and a nonredundant gene catalogue was constructed by MetaGeneMark and CD-HIT. Sequence-based gene abundance was calculated from the number of mapped reads, normalized to gene length and served as basis for subsequent analyses.

- 1. *Metagenomics taxonomic analysis*

Genes obtained from sequencing were annotated by blasting against the NCBI database using DIAMOND software. Taxonomic identification was achieved using LCA algorithm in MEGAN software. Abundance of a taxonomic specie was defined as the sum of genes annotated for that species. Gene richness was calculated by counting the number of genes in each sample. Dimension reduction analyses by the Principle Component Analysis (PCoA) on taxonomic hierarchies were performed to calculate the community diversity among samples based on the Bray-Curtis distance using R package vegan (version 2.15.3). Taxonomic differences between two groups on each hierarchical level were calculated by Metastats analysis that used Permutation test to get the P value, which was then corrected by Benjamini and Hochberg False Discovery Rate.

- 1. *Metagenomics functional annotation*

The functional annotation to Kyoto Encyclopedia of Genes and Genomes (KEGG) database was performed through the DIAMOND software. Relative abundance of KEGG Ortholog (KO) and KEGG Module were determined by the sum of genes annotated to that functional level. Bray-Curtis distance-based PCoA was used to evaluate functional diversity between samples. Differential KOs revealed by Metastats analysis were uploaded using KEGG Mapper tool (version 4.3) for enriched pathways. Network depicting connections of differential pathways via common metabolites was constructed by Cytoscape (v 3.6.1).

- 1. *Fecal and serum metabolomics profiling*

Targeted fecal metabolomics and nontargeted serum metabolomics were performed by Metabo-Profile (Shanghai, China). Processing of fecal and serum samples were carried out as previously described [9]. In detail, 10 mg lyophilized feces were homogenized with 1M NaOH and centrifuged at 16,000g at 4 °C for 20 min. The resulting supernatant was saved and residues were re-extracted with cold-methanol. After centrifugation, new supernatant was combined with the previous one as aqueous fecal extract. For serum, 100 µl sample were extracted with methanol and incubated at -20^o^C for 30 min. After centrifugation, supernatant was collected and lyophilized. Both aqueous fecal extract and serum solids were then subjected to chloroformate derivatization, which was performed by a commercially available robotic workstation (GERSTEL MPS Autosampler). To do it, fecal extract was mixed with pyridine, while serum solids were first redissolved in 1M NaOH, then mixed with methanol and pyridine. After sequential addition and blending of methyl chloroformate, chloroform and sodium bicarbonate, samples were centrifuged to collect the bottom chloroform phase that was randomly analyzed by a gas chromatography coupled to time-of-flight mass spectrometer (GC/TOFMS) (Pegasus HT, LecoCorp, USA) equipped with a (5%-phenyl)-methylpolysiloxane capillary column (Rxi-5MS, 30 m x 0.25 mm i.d., 0.25 µm film thickness; Agilent). The solvent delay time was set to 2.5 min. Temperature was raised by optimized gradient to 320°C and then held there for 2 min. The temperatures of the injection, transfer interface, and ion source were set to 270, 270, and 220°C, respectively. Electron impact ionization at m/z range of 38−650 was used. The acquisition rate was 20 spectra s^−1^. All the standards were obtained from Sigma-Aldrich (St. Louis, MO). The quality control samples were prepared following the same procedures as real samples and were injected every 14 samples to ensure reproducibility. For fecal metabolomics, raw data from GC/TOFMS analysis were exported in NetCDF format to ChromaTOF software (v4.50, Leco Co., CA, USA) and processed. Individual compound identification was achieved via comparing both MS similarity and Kovats RI distance to reference standards, using a similarity cutoff score of 70%. For serum metabolomics, the raw data generated by GC-TOF/MS were processed using XploreMET (v3.0, Metabo-Profile, Shanghai, China) that integrates JiaLib metabolite database. After data transformation, all measurements were mean-centered and scaled by the standard deviation of the observed measurements. Heatmaps with clustering showing differential metabolites were generated by Heatmapper website (<http://www.heatmapper.ca/expression/>).

- 1. *Laboratory analysis*

Plasma glucose, serum lipid panel including total cholesterol, low-density lipoprotein (LDL), high-density lipoprotein (HDL), and triglycerides were analyzed with standard laboratory techniques on a Cobas 8000 c701 Analyzer (Roche Diagnostics, Germany). HbA1c was measured on D-10 Hemoglobin Testing System (Bio-Rad, USA) using HPLC method. Serum C-peptide was detected on Cobas 8000 e602 analyzer (Roche Diagnostics, Germany) using chemiluminescence method. Plasma levels of enteroendocrine hormones, including GLP-1 (active) (Millipore Cat# EGLP-35K, RRID:AB_2737305), GLP-2 (Millipore Cat # EZGLP2-37K), and PYY (Millipore Cat# EZHPYYT-66K, RRID:AB_2910201) were analyzed by commercial ELISA kits, according to the vendor’s instruction. Plasma inflammatory cytokines were measured using the Meso Scale Discovery (MSD) U-Plex assay system with customizedly configurated panels (Meso Scale diagnostics LLC, Rockville, MD). TGF-β1 was measured by commercial ELISA kit (DAKEWE Cat # DKW12-1710).

- 1. *Statistics*

All statistical analyses were performed with GraphPad Prism, unless otherwise stated. Data are presented as either scatters, mean ± SEM, or box plot showing means with minimum and maximum values, as specified in each figure. Normal distribution of each dataset was accepted when passing both Shapiro-Wilk and Kolmogorov-Smirnov tests. Different statistical methods were chosen based on normality of as-analyzed datasets. Specifically, for comparisons between two groups with matched datapoints, either two-tailed paired t tests (for normal distribution) or Wilcoxon signed-rank tests (for non-normal distribution) was used. For comparisons between two non-matched groups, two-tailed unpaired t test or Mann-Whitney test was chosen. For comparisons among multiple groups with matched datapoints, repeated-measures one-way or two-way ANOVA were adopted for data with normal distribution, whereas Friedman test or Kruskal-Wallis test were adopted for data with non-normal distribution. Sample sizes are given in each figure legend. When there were missing values, a mixed-effects model approach was fit for repeated measures data. All repeated measures underwent Geisser-Greenhouse correction. All multiple comparisons were adjusted by the original FDR method of Benjamini and Hochberg method. P or FDR values less than 0.05 were considered statistically significant. Spearman’s correlations between metabolites and clinical parameters were calculated based on a published method [10], using pooled variables collected over all the follow-up time points.

**References**

[1] Cartwright A, Wallymahmed M, Macfarlane IA, Wallymahmed A, Williams G, Gill GV. The outcome of brittle type 1 diabetes--a 20 year study. QJM. 2011;104:575-9.

[2] Kent LA, Gill GV, Williams G. Mortality and outcome of patients with brittle diabetes and recurrent ketoacidosis. Lancet. 1994;344:778-81.

[3] Pickup J, Williams G, Johns P, Keen H. Clinical features of brittle diabetic patients unresponsive to optimized subcutaneous insulin therapy (continuous subcutaneous insulin infusion). Diabetes Care. 1983;6:279-84.

[4] Cui B, Feng Q, Wang H, Wang M, Peng Z, Li P, et al. Fecal microbiota transplantation through mid-gut for refractory Crohn's disease: safety, feasibility, and efficacy trial results. J Gastroenterol Hepatol. 2015;30:51-8.

[5] Zhang T, Lu G, Zhao Z, Liu Y, Shen Q, Li P, et al. Washed microbiota transplantation vs. manual fecal microbiota transplantation: clinical findings, animal studies and in vitro screening. Protein Cell. 2020;11:251-66.

[6] Service FJ, Molnar GD, Rosevear JW, Ackerman E, Gatewood LC, Taylor WF. Mean amplitude of glycemic excursions, a measure of diabetic instability. Diabetes. 1970;19:644-55.

[7] Atamna A, Ayada G, Akirov A, Shochat T, Bishara J, Elis A. High blood glucose variability is associated with bacteremia and mortality in patients hospitalized with acute infection. QJM. 2019;112:101-6.

[8] Battelino T, Danne T, Bergenstal RM, Amiel SA, Beck R, Biester T, et al. Clinical Targets for Continuous Glucose Monitoring Data Interpretation: Recommendations From the International Consensus on Time in Range. Diabetes Care. 2019;42:1593-603.

[9] Zhao L, Ni Y, Su M, Li H, Dong F, Chen W, et al. High Throughput and Quantitative Measurement of Microbial Metabolome by Gas Chromatography/Mass Spectrometry Using Automated Alkyl Chloroformate Derivatization. Anal Chem. 2017;89:5565-77.

[10] Bland JM, Altman DG. Calculating correlation coefficients with repeated observations: Part 2--Correlation between subjects. BMJ. 1995;310:633.

1. **Supplementary Table**

| **Table S1. Power analysis of each GV index calculated using CGM data** | | | | | | |
| --- | --- | --- | --- | --- | --- | --- |
|  | MAGE (mmol/L) | SDBG (mmol/L) | BG>11.1 (mmol/L) | LAGE (mmol/L) | MBG (mmol/L) | TIR (%) |
| Paired t test | One-tailed | | | | | |
| Type-1 error, α | 0.05 | | | | | |
| Sample size | 14 | | | | | |
| Mean of differences (T1W minus T0) | -2.77 | -1.01 | -16.97 | -3.49 | -1.59 | 17.25 |
| Standard deviation of differences | 4.36 | 1.27 | 20.49 | 4.58 | 2.16 | 21.27 |
| Effect size | 0.63 | 0.79 | 0.83 | 0.76 | 0.73 | 0.81 |
| Individual power (%) | 72 | 88 | 90 | 85 | 83 | 89 |
| Averaged power (%) | 85 | | | | | |

1. **Supplementary Figures**

**Figure S1.** **Changes of glycemic variability indices by WMT treatment. (A)** Changes of the glycemic variability index TIR at T1W calculated using CGM data. *p<0.05 by paired t test (two-tailed), n=14. **(B-F)** Relative changes of glycemic variability indices at T1W, T1M and T3M calculated using SMBG data: (B) PPGE, (C) MBG, (D) LAGE, (E) SDBG and (F) CV. ns, nonsignificant by repeated-measures one-way ANOVA corrected by Benjamini and Hochberg method, n=14.


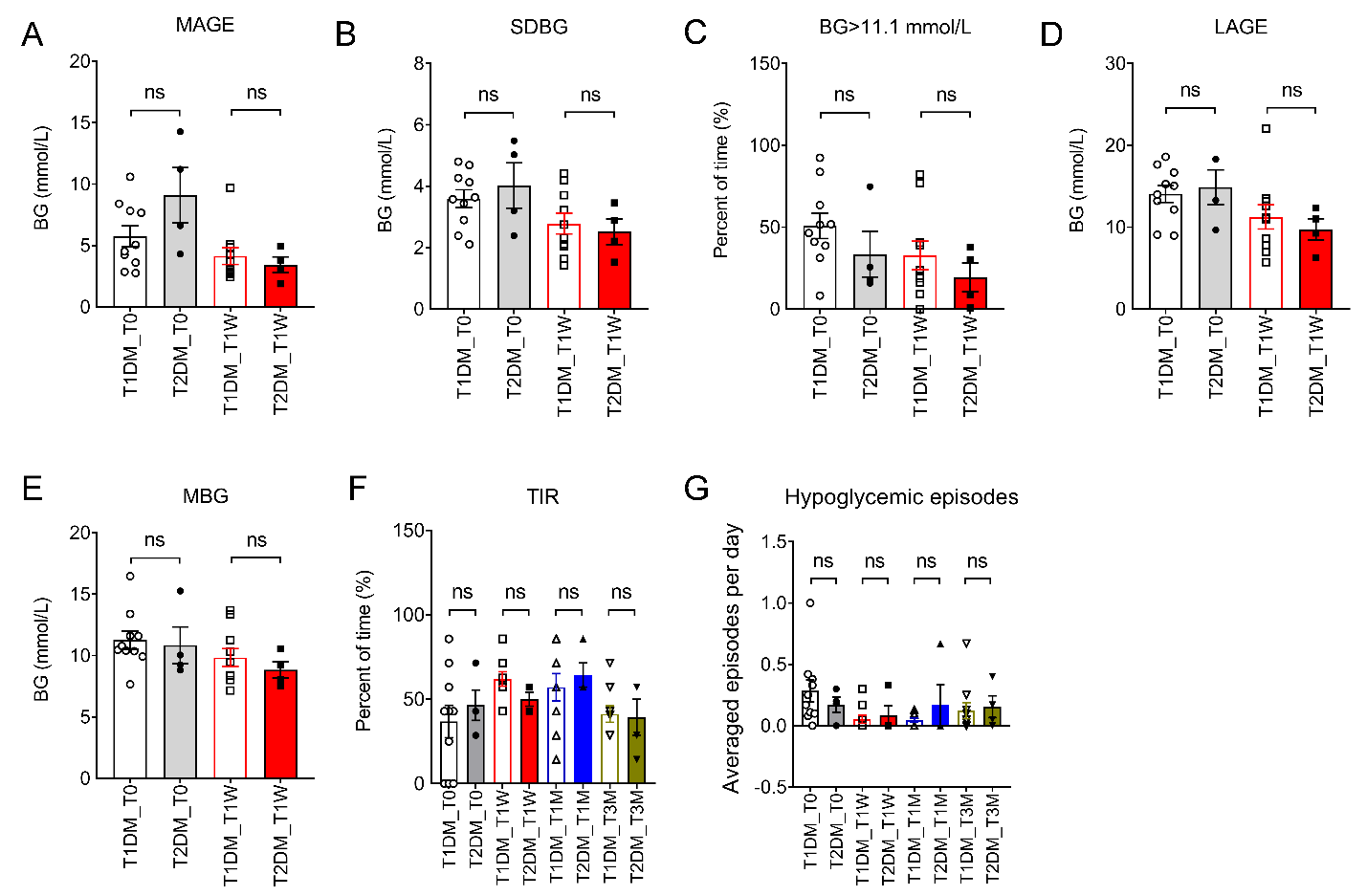


**Figure S2.** **Comparison of glycemic variability indices between T1DM and T2DM participants. (A-E)** Glycemic variability indices calculated using CGM data in participants with T1DM or T2DM at T0 and T1W: MAGE (A), SDBG (B), BG>11.1 mmol/L (C), LAGE (D) and MBG (E). **(F-G)** Glycemic variability indices calculated using SMBG data in participants with T1DM or T2DM at all follow-up time points: TIR (F) and Hypoglycemic episodes (G). ns: nonsignificant, unpaired t test (two-tailed) or Mann-Whitney test, n=10 for T1DM, n=4 for T2DM.

**
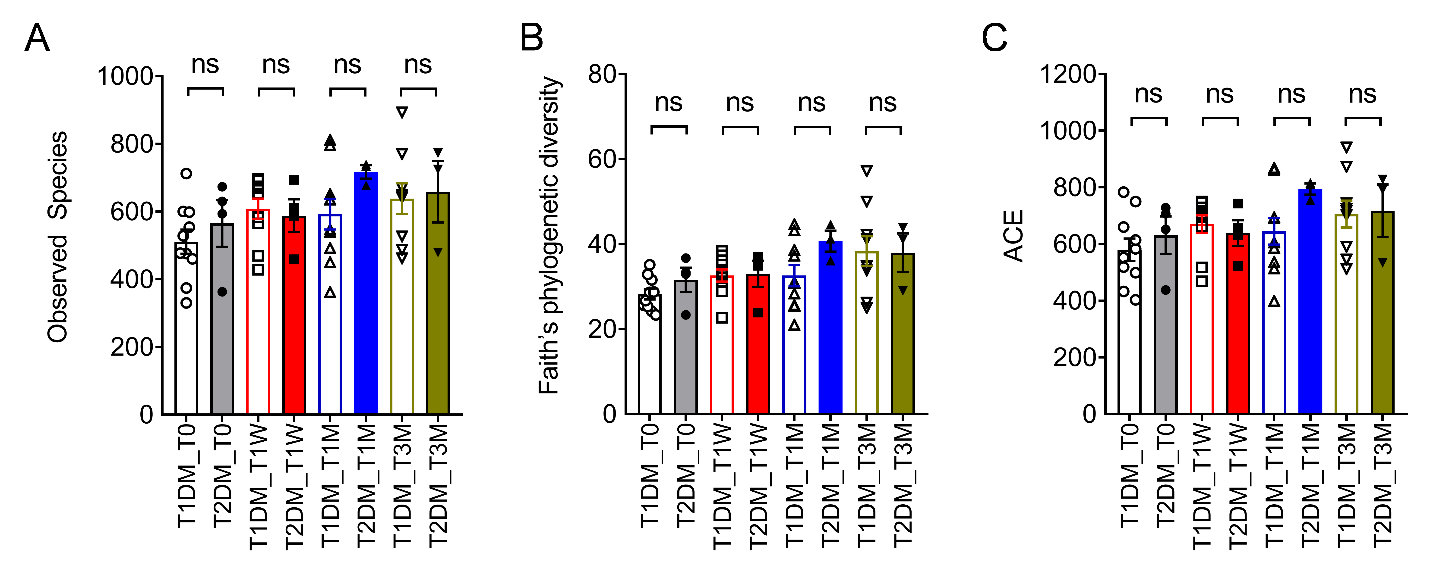
**

**Figure S3.** **Comparison of gut microbiota alpha diversity** **between T1DM and T2DM participants.** Alpha diversity at all follow-up time points, including Observed Species (A), Faith’s phylogenetic diversity (B) and ACE (C). ns: nonsignificant, unpaired t test (two-tailed) or Mann-Whitney test, n=9-10 for T1DM, n=3-4 for T2DM.


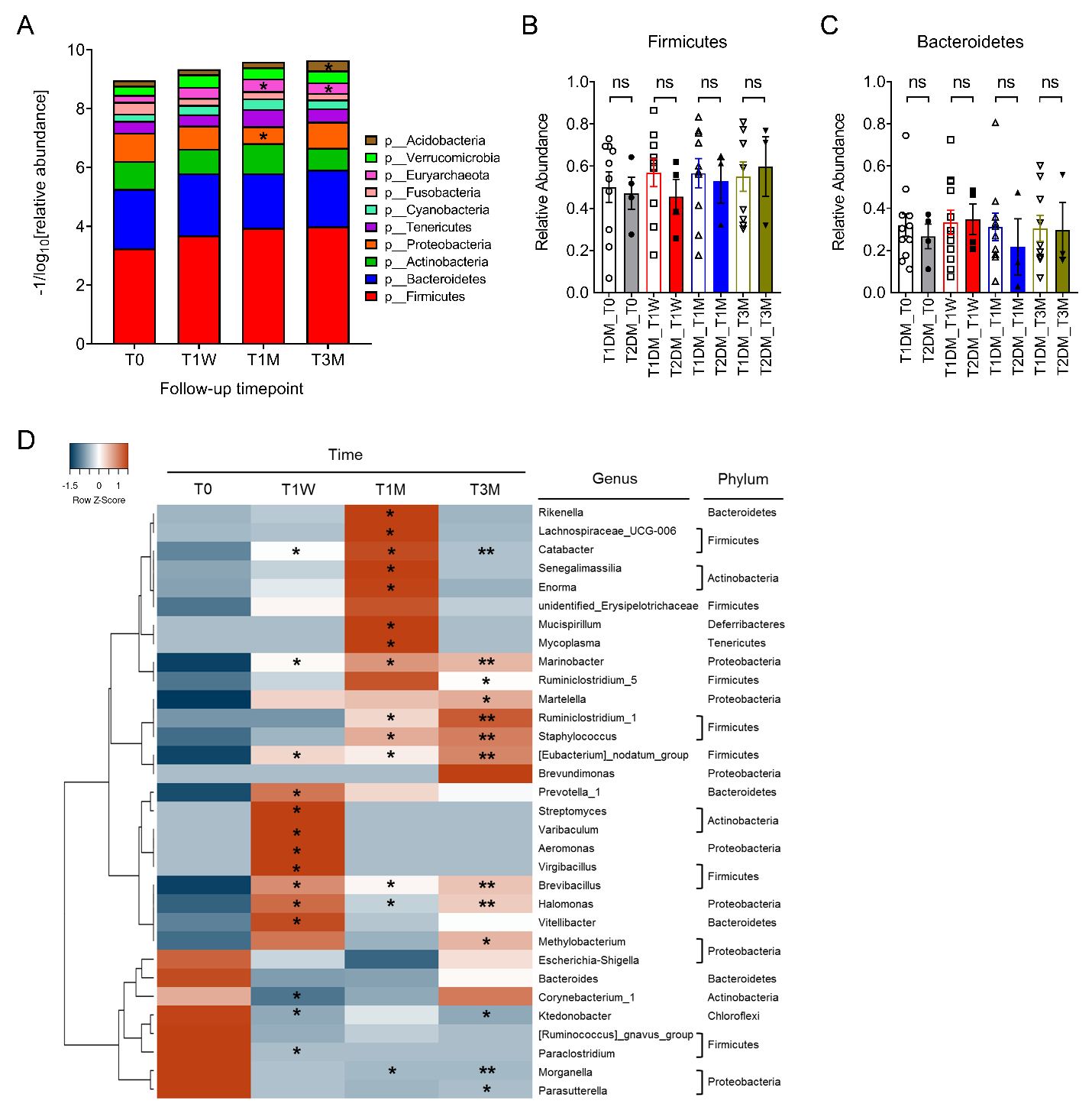


**Figure S4.** **WMT-induced alteration in gut microbiota community revealed by 16s rRNA gene sequencing.** **(A)** Bar plots of gut microbiota at phylum level. **(B-C)** Relative abundance of the phyla Firmicutes (B) and Bacteroidetes (C) at all follow-up time points between T1DM and T2DM participants. ns: nonsignificant, unpaired t test (two-tailed) or Mann-Whitney test, n=9-10 for T1DM, n=3-4 for T2DM. **(D)** Heatmap of gut microbiota at genus level. **^*^**p<0.05, **^**^**p<0.01, Metastat analysis.

**Figure S5. Changes of fasting plasma cytokines.** Bar graphs showing changes in fasting levels of plasma cytokines. FDR was calculated by a mixed-effects model for repeated measures corrected by Benjamini and Hochberg method. No statistical significance was found for each cytokine between T0 and follow-up timepoints, n=10-11.

**Figure S6. Changes of plasma hormones during SBMT.** Line charts and associated AUC analysis of plasma GLP-1 **(A)**, GLP-2 **(B)** and PYY **(C)** during SBMT performed before and after WMT intervention. Data are expressed as mean ± SEM. For each hormone, comparisons between T0 and follow-up time point (T1W or T1M) were analyzed by repeated-measures two-way ANOVA corrected by Benjamini and Hochberg method. Associated AUC was analyzed by Friedman test (GLP-1 and PYY) or repeated-measures one-way ANOVA (GLP-2), corrected by Benjamini and Hochberg method. n=9-11.
